# Characterization of FsXEG12A from the cellulose-degrading ectosymbiotic fungus *Fusarium* spp. strain EI cultured by the ambrosia beetle

**DOI:** 10.1186/s13568-020-01030-6

**Published:** 2020-05-24

**Authors:** Kiyota Sakai, Aya Yamaguchi, Seitaro Tsutsumi, Yuto Kawai, Sho Tsuzuki, Hiromitsu Suzuki, Sadanari Jindou, Yoshihito Suzuki, Hisashi Kajimura, Masashi Kato, Motoyuki Shimizu

**Affiliations:** 1 Faculty of Agriculture, Meijo University, Nagoya, Aichi 468-8502 Japan; 2 Faculty of Science and Technology, Meijo University, Nagoya, Aichi 468-8502 Japan; 3 Graduate School of Bioagricultural Sciences, Nagoya University, Nagoya, Aichi 464-8601 Japan

**Keywords:** *Fusarium* dieback, Ambrosia beetle, *Fusarium* spp., Glycoside hydrolase family 12, Enrichment culture

## Abstract

Despite the threat of *Fusarium* dieback posed due to ambrosia fungi cultured by ambrosia beetles such as *Euwallacea* spp., the wood-degradation mechanisms utilized by ambrosia fungi are not fully understood. In this study, we analyzed the *16S rRNA* and *18S rRNA* genes of the microbial community from the *Ficus* tree tunnel excavated by *Euwallacea interjectus* and isolated the cellulose-degrading fungus, *Fusarium* spp. strain EI, by enrichment culture with carboxymethyl cellulose as the sole carbon source. The cellulolytic enzyme secreted by the fungus was identified and expressed in *Pichia pastoris*, and its enzymatic properties were characterized. The cellulolytic enzyme, termed FsXEG12A, could hydrolyze carboxymethyl cellulose, microcrystalline cellulose, xyloglucan, lichenan, and glucomannan, indicating that the broad substrate specificity of FsXEG12A could be beneficial for degrading complex wood components such as cellulose, xyloglucan, and galactoglucomannan in angiosperms. Inhibition of FsXEG12A function is, thus, an effective target for *Fusarium* dieback caused by *Euwallacea* spp.

## Introduction

*Euwallacea* spp. is a genus of ambrosia beetles distributed over Asia into Israel, Central America, and in at least five different locations within the United States. These beetles penetrate wood packaging and plant material (Haack 2006; Kirkendall and Ødegaard 2007; O’Donnell et al. 2015; Ploetz et al. 2016; Wingfield et al. 2010). Ambrosia beetles including *Euwallacea* spp. carry fungi in specialized structures on their integument called mycangia. Symbiotic fungi (consistent associates) often include only two to three partners per ambrosia beetle species. *Euwallacea* spp. is a genus of over 40 species within the *Xyleborini* and is the beetle genus known to cultivate ambrosia fusaria, as their larvae feed on the ectosymbiotic filamentous fungus (Gadd and Loos 1947). *Euwallacea* spp. and their symbiont ambrosial fusaria largely colonize wood from dead or declining species from at least 48 plant families (Aoki et al. 2019; Danthanarayana 1968; Hulcr et al. 2007). They are also known as destructive pests of several economically important woody plants, including Chinese tea (*Camellia sinensis* L. Kuntze), avocado (*Persea americana* Mill.), citrus (*Citrus* spp.), and cacao (*Theobroma cacao* L.), where they can cause extensive dieback and even death (Brayford 1987). *Fusarium* dieback is known to be responsible for serious damage mainly to avocado, box elder (*Acer negundo* L.), castor bean (*Ricinus communis* L.), and English oak (*Quercus robur* L.) (Mendel et al. 2012). Recently, the evolutionary histories of key representatives of the *Fusarium* and *Euwallacea* clades were reconstructed (O’Donnell et al. 2015). *Fusarium* spp., termed AF 1–12, were identified, and the dominant fungal symbiont of *Euwallacea interjectus* was a specialized ambrosia fungus, *Fusarium* sp. strain AF-3 (O’Donnell et al. 2015).

The effects of symbiotic fungi on the ambrosia beetle host vary from beneficial to neutral to negative (Klepzigl and Six 2004). Most studies have identified these interactions as obligate mutualisms in which the ambrosia beetles rely on nutritional supplementation from the fungi, and the fungi easily colonize the targeted host trees through the beetle’s transport. The ability of ambrosia fungi to degrade and assimilate wood components (e.g. cellulose and hemicellulose) allows for nutritional supplementation to the ambrosia beetles (De Fine Licht and Biedermann 2012). Few studies have conducted a microbial analysis of ambrosia fungi using the denaturing gradient gel electrophoresis (DGGE) method. Despite the threat posed by ambrosia fungi cultured by ambrosia beetles such as *Euwallacea* spp., the wood-degradation mechanisms employed by ambrosia fungi are not fully understood.

In this study, we analyzed the *16S rRNA* and *18S rRNA* genes of the microbial community from the *Ficus* tree tunnel excavated by *Euwallacea interjectus*. Furthermore, we isolated a cellulose-degrading fungus, termed *Fusarium* spp. strain EI, by enrichment culture with carboxymethyl cellulose (CMC) as the sole carbon source. The cellulolytic enzyme secreted by the fungus was also identified, expressed in *Pichia pastoris*, and its enzymatic properties were characterized.

## Materials and methods

### Specimens

**Fig. 1:**
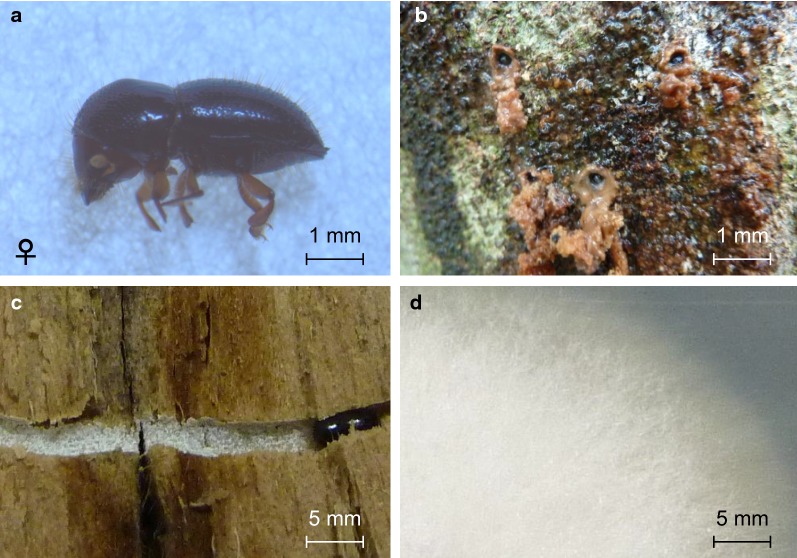
Photographs of fungus cultivation by *Euwallacea interjectus* in *Ficus carica.***a** Adult female *E. interjectus*. **b** Entrance holes of *E. interjectus* boring into the *F. carica* trunk. **c** Ambrosia fungi on the walls of a tunnel excavated by *E. interjectus*. **d** The fungal mycelia grown on a PDA plate

Logs of *Ficus carica* attacked and bored by *E. interjectus* (Fig. 1a–c) were sampled from fig orchards in Tokoname, Aichi, Japan (latitude: 35° 11′; longitude: 136° 54′) on 30 September 2009. Tunnels excavated by *E. interjectus* (mother beetle) (Fig. 1a) were carefully opened to expose the inner surface of the cavities, on which ambrosia fungi were cultivated as larval food (Fig. 1d). Female adults (daughters) emerging from the logs were reared on semi-artificial diets with a two-layer structure as described by Mizuno and Kajimura (2009) to obtain successive generations (Mizuno and Kajimura 2009).

### Chemicals and reagents

CMC, xyloglucan, lichenan, curdlan, laminarin, pustulan, glucomannan, and galactomannan were purchased from Megazyme International (Bray, Ireland). MCC was obtained from Funakoshi (Tokyo, Japan).

### Polymerase chain reaction (PCR)-DGGE analysis

The microbial community in the tunnel excavated by *E. interjectus* was harvested by centrifugation at 8000×*g* for 5 min. The harvested cells were suspended in saline (150 mM NaCl, 10 mM Tris–HCl; pH 8.0) and centrifuged again at 8000×*g* for 5 min. The total DNA of the microbial community was extracted using an FTA Elute card (GE Healthcare, Waukesha, WI, USA). The *16S/18S rRNA* genes were amplified using universal primers (Table 1), as described previously (May et al. 2001; Muyzer et al. 1993). Each amplification reaction mixture (50 μL) consisted of 1 µL of KOD-Plus-Neo DNA polymerase (Toyobo Co., Ltd.; Osaka, Japan), 5 μL of 5× KOD buffer, 5 μL of dNTPs, 1.5 μL of forward and reverse primer (10 µM), 3 μL of template DNA (100 ng), 3 μL of MgSO_4_ solution (25 mM), and 30 μL of ddH_2_O. PCR was implemented as follows: after an initial denaturation at 94 °C for 2 min, 25 cycles at 94 °C for 15 s, 50 °C for 30 s, and 72 °C for 30 s, with a final extension at 72 °C for 5 min were performed. The PCR amplicons were purified using a QIAquick PCR Purification Kit (Qiagen, Hilden, Germany), and the DNA concentration was determined by measuring the absorbance at 260 nm. The electrophoresis of the amplification products was performed in 7% (w/v) polyacrylamide gel (37.5:1 acrylamide:bis-acrylamide) in the presence of a linear denaturing gradient that ranged from 40 to 70% using a DCode Mutation Detection System (Bio-Rad, Hercules, CA, USA). DNA bands were detected using a blue light transilluminator (Thermo Fisher Scientific, MA, USA) and were carefully excised. DNA was extracted from the gel pieces by incubation in 50 µL ddH_2_O for 24 h at 4 °C and then sequenced.

**Table 1:** Oligonucleotide primers used in this study

Primer	Gene	Nucleotide sequence^a^
DGGE analysis		
16S-f	*16S rRNA*	5′-CGCCCGGGGCGCGCCCCGGGCGGGGCGGGGGCACGGGGGGAACGCGAAGAACCTTAC-3′
16S-r	*16S rRNA*	5′-CGGTATGTACAAGGCCCGGGAACG-3′
18S-f	*18S rRNA*	5′-CGCCCGCCGCGCCCCGCGCCCGGCCCGCGGCCCCCGCCCCATTCCCCGTTACCCGTTG-3′
18S-r	*18S rRNA*	5′-GTAGTCATATGCTTGTCTC-3′
EF-f	*EF*-*1α*	5′-ATGGGTAAGGAAGACAAGAC-3′
EF-r	*EF*-*1α*	5′-GGAAGTACCAGTGATCATG-3′
Cloning primers for the 25-kDa protein gene		
25-kDa-f	*FsXEG_17404*	5′-ATGAAGGGCTCTCTTGTCTTC-3′
25-kDa-r	*FsXEG_17404*	5′-TTAGTTGACGTGAGCATTGAA-3′
Plasmid construction for recombinant protein production		
17404-f	*FsXEG_17404*	5′-AGAAAAGAGAGGCTGAAGCTGAATTCCAGTCTCTCTGCGACCAATA-3′
17404-r	*FsXEG_17404*	5′-GACGGCCGGCTGGGCCACGTGAATTCTTAGTTGACGTGAGCATTGA-3′

^a^GC-clamps are underlined. Primers for *16S rRNA* and *18S rRNA* were designed according to Muyzer et al. (1993) and May et al. (2001), respectively

### Isolation and identification of cellulose-degrading microorganisms by enrichment culture technique

The microorganisms from the tunnel excavated (Fig. 1c) by *E. interjectus* were collected and then cultured at 28 °C in MM liquid medium (10 mM NaNO_3_, 10 mM KH_2_PO_4_, 7 mM KCl, 2 mM MgSO_4_, 2 mL/L Hutner’s trace metals, 1.5% agar; pH 6.5) containing 1.0% CMC as the sole carbon source. In addition, *E. interjectus* (Fig. 1a) collected from the excavated tunnels in *Ficus* trees was sprayed and sterilized with 70% ethanol solution. Then, the heads including the mycangia were cut off and aseptically disrupted with a mortar and pestle. The symbiotic microorganisms were also cultured at 28 °C in MM liquid medium containing CMC. After 5 days of incubation, 1 mL of culture supernatant was added to 50 mL of fresh cellulose medium and then cultured at 28 °C. This operation was repeated four times in total. After enrichment culture using MM liquid medium containing CMC, 10 µL of culture supernatant was applied to MM agar medium containing CMC at 28 °C, and then the cellulose-degrading microorganism was isolated. The 18S rRNA and the elongation factor-1α (*EF*-*1α*) genes from the isolated fungus from the excavated tunnel and *E. interjectus* were sequenced using the primers shown in Table 1. *Fusarium* spp. strain EI has been deposited as NBRC number 113538 in the International Patent Organism Depository, National Institute of Technology and Evaluation (Tokyo, Japan).

### Zymogram analysis of extracellular cellulolytic enzymes

*Fusarium* spp. strain EI was cultured on MM agar medium containing 1.0% CMC at 37 °C for 4 days, and the conidia were harvested. The conidia (1 × 10^8^) were inoculated and cultured in liquid MM medium containing 1.0% CMC as the sole carbon source at 28 °C for 0–6 days. The culture supernatants were concentrated using an Amicon Ultra filter unit (Merck-Millipore, Billerica, MA, USA) and dialyzed against 50 mM Tris–HCl buffer (pH 8.0). All protein collection steps were performed at 4 °C.

To identify the extracellular cellulolytic enzymes from *Fusarium* spp. strain EI, zymography was performed. *Fusarium* spp. strain EI was cultured in liquid MM medium containing 1.0% CMC at 28 °C for 3 days, at which time the extracellular proteins secreted from *Fusarium* spp. strain EI were concentrated by trichloroacetic acid (TCA) treatment, and TCA precipitates were washed with acetone and dried. The concentrated proteins were separated by sodium dodecyl sulfate-polyacrylamide gel electrophoresis (SDS-PAGE) and zymogram gels. Zymogram analysis was performed by co-polymerising 0.5% CMC with 12% polyacrylamide gel. After electrophoresis, the gel was soaked for 1 h in 2.5% (v/v) Triton X-100 to remove the SDS and refold the proteins in the gel. The gel was then thoroughly washed three times in MilliQ water and incubated at 30 °C for 60 min in 50 mM acetate buffer (pH 4.0). The gel was stained with 0.1% (w/v) Congo red solution for 30 min. To detect enzyme activity, the gel was de-stained with 1 M of NaCl until pale-red hydrolysis zones appeared against a red background.

### Identification of cellulolytic enzymes from *Fusarium* spp. strain EI

The protein band with cellulose-degrading activity was excised from the gel, digested with trypsin, and analyzed using matrix assisted laser desorption ionisation-time of flight tandem mass spectrometry (MALDI-TOF/TOF–MS), as previously described (Shimizu et al. 2015). Peptide mass fingerprints and MS/MS spectra were analyzed using the MASCOT search engine (Matrix Science Ltd., London, UK) against the database of all entry proteins, as previously described (Sakai et al. 2017a, b).

### Cloning of the *GH12* gene from *Fusarium* spp. strain EI

*Fusarium* spp. strain EI was cultured in MM medium containing CMC as the sole carbon source for 3 days as described above, and total RNA was prepared by using the RNeasy Mini Kit (Qiagen, Venlo, The Netherlands) per the manufacturer’s instructions. Single-strand cDNA was synthesized from total RNA extracted from disrupted fungal cells, as described previously (Shimizu et al. 2009; Sakai et al. 2018). Complementary DNA fragment encoding the GH12 protein was amplified using the primers shown in Table 1. The PCR product was cloned in pPICZα-A (Novagen, Darmstadt, Germany), and the deduced nucleotide sequence was determined using an automated DNA sequencer (CEQ 2000, Beckman Coulter, Brea, CA, USA).

### Preparation of recombinant protein

Complementary DNA fragments encoding the GH12 protein gene were prepared by PCR using the oligonucleotide primer set shown in Table 1. The PCR product was digested by EcoRI and then ligated into pPICZα-A (Novagen, Darmstadt, Germany) that had been digested with the same restriction enzyme. The resulting plasmid was introduced into *P. pastoris* KM71H (Invitrogen, Carlsbad, CA), and the resulting strain was cultured to produce recombinant GH12 protein, as previously described (Kamijo et al. 2019). The culture supernatant was concentrated using an Amicon Ultra filter unit (Merck-Millipore, Massachusetts, USA). The concentrated protein solution was fractionated on a HiTrap Q column (GE Healthcare, Chicago, USA) using a linear gradient of 0–0.5 M NaCl in 50 mM Tris–HCl (pH 8.0). The elution fraction was concentrated using an Amicon Ultra filter unit (Merck-Millipore, Massachusetts, USA). The purified enzyme was cleaved with Endoglycosidase H (New England Biolabs, Ipswich, MA, USA) to remove N-linked glycans according to the manufacturer’s instructions. Deglycosylated protein was applied to a Superose 6 10/300 GL column (GE Healthcare, Chicago, USA), and recombinant protein was eluted with 50 mM Tris–HCl (pH 8.0) containing 50 mM NaCl and dialyzed against 50 mM Tris–HCl (pH 8.0). All protein purification steps were conducted at 4 °C. Protein concentrations were assayed using the Bradford Protein Assay (Bio-Rad Laboratories, California, USA) with bovine serum albumin as the standard.

### Enzyme assays

The glycoside hydrolase activity was assayed in 0.5 mL reaction mixtures containing 50 mM acetate buffer (pH 4.0), 0.2–5% (w/v) substrates, and purified recombinant protein. Reactions were incubated at 50 °C, and GH12 protein was removed from the reaction solution using a Nanosep^®^ Centrifugal Device (Pall Corporation, Port Washington, NY, USA), as described in the instruction manual. Flow-through fractions were boiled at 100 °C for 30 min, and the reducing sugars produced by the recombinant protein were measured using 3,5-dinitrosalicylic acid (DNS), as described previously (Sakai et al. 2017a, b). Standard curves were prepared based on solutions containing various concentrations of glucose. One unit of glycoside hydrolase activity was defined as the amount of enzyme required to produce 1 µmol of reducing sugar (glucose equivalents) per minute.

The reaction products released from polysaccharides were separated on TLC Silica gel 60 plates (Merck-Millipore, Massachusetts, USA) using *n*-butanol:ethanol:water (10:8:7), visualized by staining with 0.82% (v/v) *N*-(1-naphthyl) ethylenediamine dihydrochloride and 8.6% (v/v) sulfuric acid in ethanol, and baked at 105 °C for 5 min. The reaction products released from xyloglucan and lichenan were analyzed using MALDI-TOF–MS as previously described (Shimizu et al. 2015).

### pH stability and thermostability of purified FsXEG12A

The pH stability and thermostability of the GH12 enzyme (i.e. the FsXEG12A) were determined using 1.0% xyloglucan as a substrate. FsXEG12A (1.0 μM) was incubated in 50 mM glycine–HCl (pH 1.0–3.0), 50 mM sodium acetate (pH 3.0–5.0), 50 mM sodium phosphate (pH 5.0–7.0), and 50 mM Tris–HCl (pH 7.0–10.0) at 4 °C for 24 h, and then the residual xyloglucanase (XEG) activity against 1.0% xyloglucan was measured at the optimal pH of 4.0 at 50 °C for 60 min. Purified FsXEG12A was also incubated at temperatures ranging from 40 to 60 °C for 60 min. Residual FsXEG12A activity was assayed at the optimal pH at 50 °C for 60 min. The amount of reducing sugar produced by FsXEG12A was measured using the DNS method (Sakai et al. 2017a, b).

### Accession number(s)

The *16S rRNA* gene sequences of the bacteria in the excavated tunnel by *E. interjectus* were deposited in the GenBank database under Accession numbers LC537448 to LC537462, respectively. The *18S rRNA*, *EF*-*1α*, and *FsXEG12A* gene sequences of the fungus isolated from the excavated tunnel by *E. interjectus* were deposited in the GenBank database under Accession numbers LC534254, LC534255, and LC634256, respectively.

### Bioinformatics

Nucleotide and amino acid sequences were aligned using ClustalW (Thompson et al. 1994). Phylogenetic analyses of full-length amino acid sequences were conducted via the neighbor-joining method using MEGA 7 software (Tamura et al. 2007).

## Results

### DGGE profile of tunnels excavated by *E. interjectus*

**Fig. 2:**
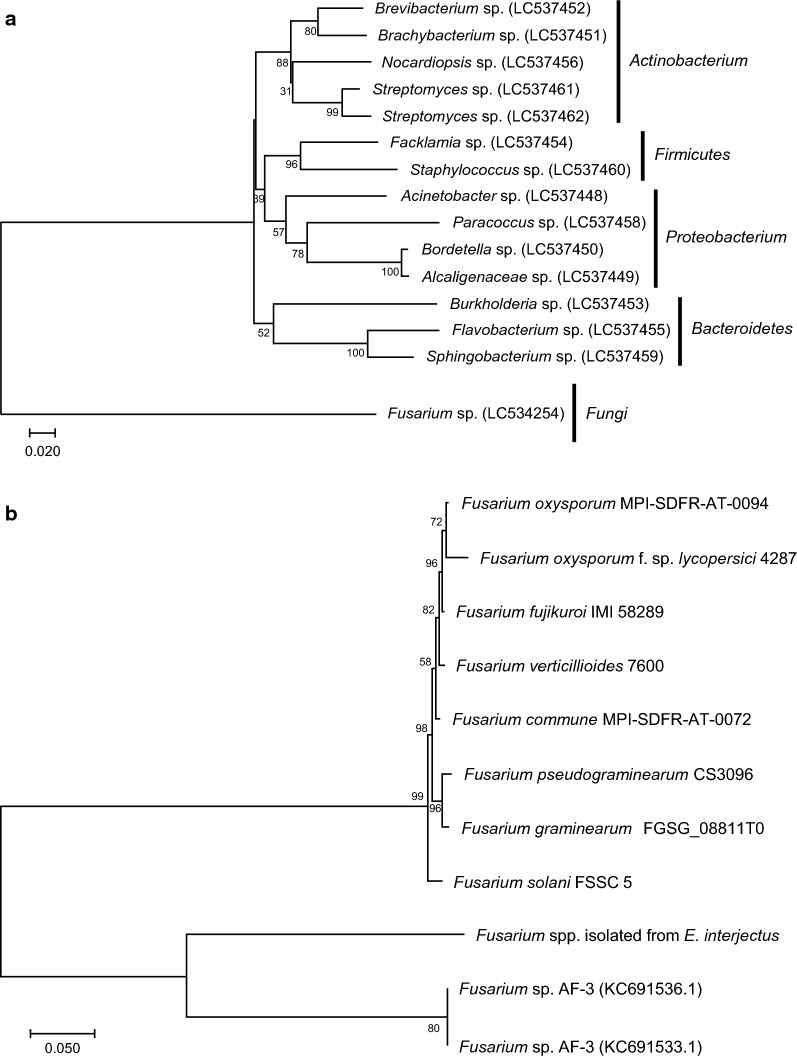
Phylogenetic tree analysis of DGGE profiles of *Fusarium* isolated from *Ficus* tree tunnel. **a** Phylogenetic tree of *16S rRNA* and *18S rRNA* gene sequences obtained from DGGE analysis. The *16S rRNA* gene sequences of the bacteria and the *18S rRNA* gene sequence of the fungus in the excavated tunnel by *E. interjectus* were registered in the GenBank database under Accession numbers LC537448 to LC537462, and LC534254, respectively. The microbial DNA sequences were identified using the NCBI database. **b** Phylogenetic tree of the partial *EF*-*1α* gene sequences of *Fusarium* spp. isolated from a tunnel in a *Ficus* tree excavated by *E. interjectus*, various *Fusarium* species registered in the JGI database (https://mycocosm.jgi.doe.gov/Fusarium/Fusarium.info.html), and *Fusarium* sp. AF-3 in the NCBI database. *EF*-*1α* genes of KC691533.1 from *Fusarium* sp. AF-3, KC691536.1 from *Fusarium* sp. AF-3, 599354 from *F. commune* MPI-SDFR-AT-0072, 8475 from *F. fujikuroi* IMI 58289, 9400 from *F. graminearum* FGSG_08811T0, 17733 from *F. oxysporum* f. sp. *lycopersici* 4287, 554906 from *F. oxysporum* MPI-SDFR-AT-0094, 4767 from *F. pseudograminearum* CS3096, 485664 from *F. solani* FSSC 5, 10379 from *F. verticillioides* 7600, and LC534255 from *Fusarium* spp. strain EI are shown. The phylogenetic tree was constructed using the neighbor-joining method with 1000 bootstrap replicates

The body length of the adult female *E. interjectus* was 4–5 mm (Fig. 1a). *E. interjectus* penetrated the *Ficus carica* tree (Fig. 1b) and subsequently cultivated ambrosia fungi, which were present in the mycangia or on the exoskeleton. The total DNA was directly extracted from the microbial community in the tunnels excavated by *E. interjectus* (Fig. 1c, d), and the sequences of *16S rRNA* and *18S rRNA* genes were amplified using the primer sets shown in Table 1. The DGGE bands of *16S rRNA* and *18S rRNA* genes were sequenced (Additional file 1: Table S1) and identified using the NCBI database (Table 2). Identified bacteria were categorized into four classes: (1) *Brevibacterium* sp. (NR_118221.1, 100% identity), *Brachybacterium* sp. (KX989329.1, 100% identity), *Nocardiopsis* sp. (MG597576.1, 100% identity), and *Streptomyces* spp. (KY015030.1, 96%, 99% identity) belonging to *Actinobacterium*; (2) *Facklamia* sp. (KU747974.1, 97% identity) and *Staphylococcus* sp. (KF911124.1, 97% identity) belonging to *Firmicutes*; (3) *Acinetobacter* sp. (CP018259.1, 100% identity), *Paracoccus* sp. (KT345705.1, 98% identity), *Bordetella* sp. (AF227829.1, 99% identity), and *Alcaligenaceae* sp. (MF182113.1, 99% identity) belonging to *Proteobacterium*; and (4) *Burkholderia* sp. (KM974662.1, 98% identity), *Flavobacterium* sp. (MF405100.1, 99% identity), and *Sphingobacterium* sp. (KF911124.1, 97% identity) belonging to *Bacteroides* (Table 2 and Fig. 2a). The identified fungus belonged to *Fusarium* spp. (MF522223.1, 99% identity) (Table 2, Fig. 2a and Additional file 1: Fig. S1).

**Table 2:** Species identification with the *16S rRNA* and *18S rRNA* genes from the total microbial community in the tunnel excavated by *Euwallacea interjectus*

Accession number	Closest relative species in BLAST analysis	Identity scores %^a^	Kingdom
LC537448	*Acinetobacter* sp.	100% (CP018259.1)	*Bacteria*
LC537449	*Alcaligenaceae* sp.	99% (MF182113.1)	
LC537450	*Bordetella* sp.	99% (AF227829.1)	
LC537451	*Brachybacterium* sp.	100% (KX989329.1)	
LC537452	*Brevibacterium* sp.	100% (NR_118221.1)	
LC537453	*Burkholderia* sp.	98% (KM974662.1)	
LC537454	*Facklamia* sp.	97% (KU747974.1)	
LC537455	*Flavobacterium* sp.	99% (MF405100.1)	
LC537456	*Nocardiopsis* sp.	100% (MG597576.1)	
LC537457	*Ochrobactrum* sp.	100% (MF928349.1)	
LC537458	*Paracoccus* sp.	98% (KT345705.1)	
LC537459	*Sphingobacterium* sp.	97% (KF911124.1)	
LC537460	*Staphylococcus* sp.	100% (EU629159.1)	
LC537461	*Streptomyces* sp.1	96% (KY015030.1)	
LC537462	*Streptomyces* sp.2	99% (KY015030.1)	
LC534254	*Fusarium* spp.	99% (MF522223.1)	*Fungi*

^a^Identity represents the sequence identity (%) compared with the *16S rRNA* or *18S rRNA* gene sequences registered in the GenBank database

### Isolation and identification of the cellulose-degrading fungus

The microorganisms from the tunnel excavated by *E. interjectus* (Fig. 1c) were cultured with CMC as the sole carbon source. All *18S rRNA* sequences of 10 grown colonies (Additional file 1: Fig. S1) were the same, and sequences of the *EF*-*1α* genes, which have been used for DNA typing to resolve all known species within the Ambrosia *Fusarium* clade (Kasson et al. 2013), were located near that of *Fusarium* sp. strain AF-3 in the phylogenetic tree (Fig. 2b and Additional file 1: Fig. S2). The fungus isolated in the current study was a species related to *Fusarium* sp. strain AF-3 and was previously reported to be a symbiotic fungus with *E. interjectus* (O’Donnell et al. 2015). Therefore, we named this novel strain *Fusarium* spp. strain EI. The *18S rRNA* sequence of *Fusarium* spp. strain EI corresponded to that of *Fusarium* spp. as identified above in the DGGE analysis (Table 2 and Additional file 1: Fig. S1). Isolation of fungi from *E. interjectus* (Fig. 1a) was also performed, and only *Fusarium* spp. strain EI was isolated using MM liquid medium containing CMC. These results suggested that *Fusarium* spp. strain EI was a cellulose-degrading symbiotic fungus found in *E. interjectus*.

### Identification of the cellulolytic enzyme produced by *Fusarium* spp. strain EI

**Fig. 3:**
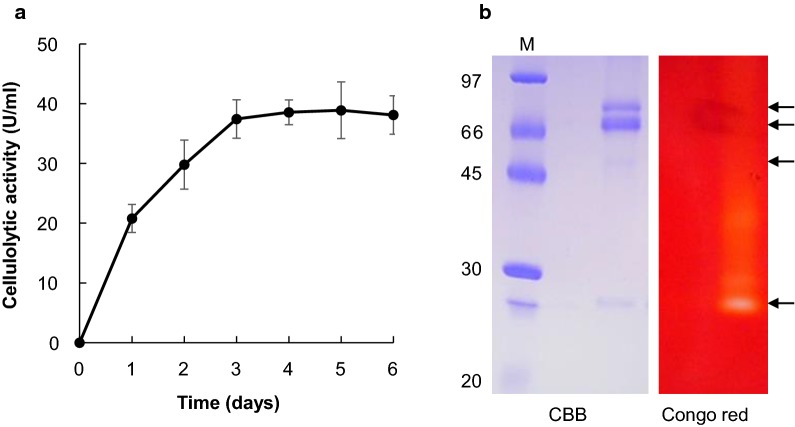
Time course of cellulase activity in the culture supernatant and zymography analysis of the extracellular proteins. **a** Cellulolytic activity in culture supernatants from *Fusarium* spp. strain EI grown with 1.0% CMC for 0–6 days. Data are presented as mean ± standard deviation of three experiments. **b** Extracellular proteins produced by *Fusarium* spp. strain EI during a 3-day incubation resolved on SDS-PAGE (left panel) and zymogram gels (right panel). The detected protein bands on SDS-PAGE are indicated by arrows

A time-course of cellulolytic activity in the culture supernatant of *Fusarium* spp. strain EI grown in MM medium containing CMC as the sole carbon source was obtained. Cellulase activity gradually increased, reaching a maximum of 37.4 U/mL at 3 days (Fig. 3a). The extracellular cellulolytic enzymes produced by *Fusarium* spp. strain EI were analyzed by SDS-PAGE and zymogram gels using CMC as the substrate (Fig. 3b). Four major protein bands were observed on SDS-PAGE, whereas one of the four protein bands was detected when determining cellulolytic activity by zymographic analysis of the culture supernatants (Fig. 3b). A protein band of approximately 25-kDa with cellulolytic activity was identified as EMT67806.1 by peptide mass fingerprinting and MS/MS spectrum analysis using MALDI-TOF/TOF–MS (Fig. 3b and Additional file 1: Table S1). These results indicate that *Fusarium* spp. strain EI predominantly secretes a cellulolytic enzyme with high similarity to EMT67806.1 in liquid MM medium containing 1.0% CMC.

### Cloning, expression, and purification of the 25-kDa protein

**Fig. 4:**
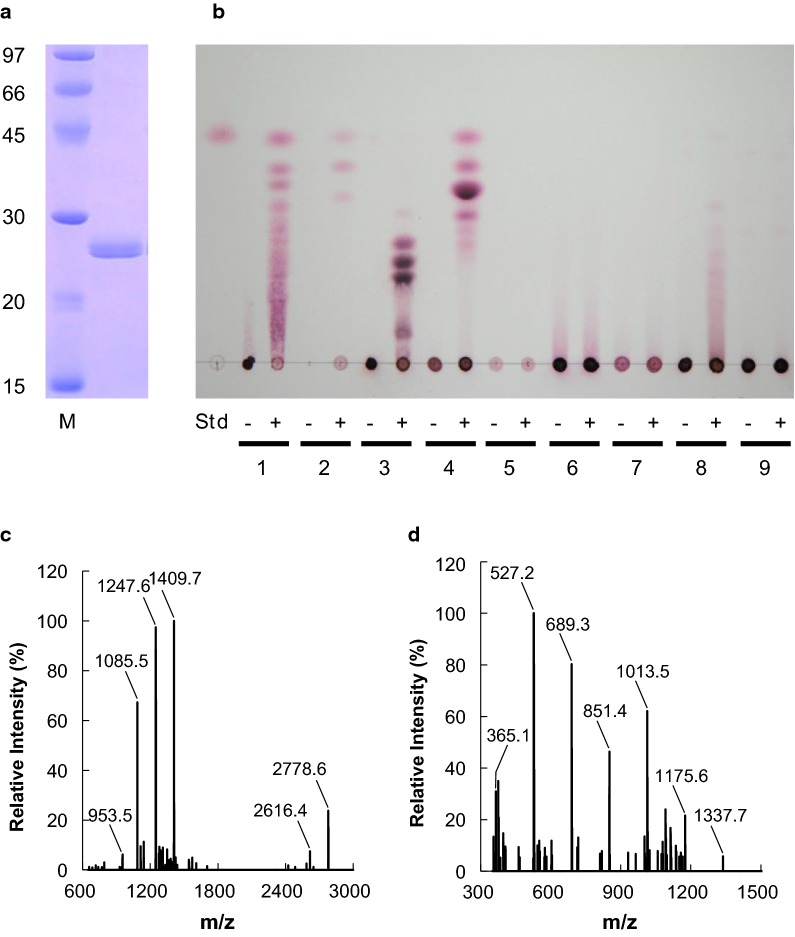
SDS-PAGE of recombinant GH12 enzyme, and TLC and MALDI-TOF–MS analyses of reaction products. **a** Purified enzyme (1 μg) resolved by SDS-PAGE stained with Coomassie brilliant blue. Lane 1, GH12 enzyme deglycosylated by EndoH; Lane M, protein molecular mass makers. **b** TLC analysis of soluble products in reaction mixtures with (+) and without (−) enzyme using CMC (lane 1), MCC (lane 2), xyloglucan (lane 3), lichenan (lane 4), curdlan (lane 5), laminarin (lane 6), pustulan (lane 7), glucomannan (lane 8), or galactomannan (lane 9) as the substrate. GH12 enzyme was incubated with 1.0% substrate in 50 mM acetate buffer (pH 3.0) at 50 °C for 60 min. **c**, **d** Reaction products from xyloglucan (**c**) and lichenan (**d**) were identified using MALDI-TOF–MS

For cloning of the full-length DNA encoding the cellulolytic enzyme from *Fusarium* spp. strain EI, we designed the forward and reverse primers (25-kDa-f and 25-kDa-r) based on the DNA sequence of EMT67806.1 (https://www.uniprot.org/uniprot/N1RQF4#expression). The DNA sequence contained an open reading frame (714 bp) encoding 238 amino acids (Additional file 1: Fig. S3). The N-terminus (amino acids 1–18) was a putative signal peptide sequence. The putative amino acid sequence resembled the sequences of the following annotated proteins: murein transglycosylase from *F. oxysporum* f. sp. *lycopersici* 4287 (XP_018258392.1, 100%), probable endoglucanase I precursor from *F. proliferatum* (CVL13720.1, 97%), murein transglycosylase from *F. verticillioides* 7600 (XP_018760412.1, 94%), and endoglucanase-1 precursor from *F. graminearum* PH-1 (XP_011325323.1, 83%). These results showed that the cellulolytic enzyme produced by *Fusarium* spp. strain EI corresponds to a 25-kDa protein belonging to the glycoside hydrolase family 12 (GH 12). To examine the function of the GH 12 enzyme in detail, it was expressed in *P. pastoris* (Fig. 4a). For the *Pichia* expression system, the forward primer without the signal peptide and the reverse primer were designed as 17404-f and 17404-r, respectively (Table 1).

### Substrate specificity of the recombinant GH12 enzyme

The substrate specificity of recombinant FsXEG12A was investigated. The GH 12 enzyme hydrolyzed CMC, microcrystalline cellulose (MCC), xyloglucan, lichenan, and glucomannan, while no hydrolase activity was detected towards curdlan, laminarin, pustulan, and galactomannan (Fig. 4b). The reaction products generated by recombinant FsXEG12A from xyloglucan and lichenan were analyzed using MALDI-TOF–MS (Fig. 4c, d). Xyloglucan consists of repeating backbone tetramers of XXXG, XXLG (or XLXG) and XLLG, where G is an unbranched β-d-glucopyranose (β-d-Glc*p*) residue, X is a β-d-Glc*p* residue that is decorated by α-1,6-d-xylopyranose (α-d-Xyl*p*), and L is a β-d-Glc*p* residue with a β-d-galactopyranose (β-d-Gal*p*)-(1 **→ **2)-α-d-Xyl*p* branch. The main product peaks are consistent with the masses of sodium adducts of XXXG (*m/z* 1085), XXLG or XLXG (*m/z* 1247), and XLLG (*m/z* 1409), and the peaks correspond to sodium adducts of octameric subunits (*m/z* 2615 and 2778) (Fig. 4c). Similar spectra of the reaction products have been reported previously (Bauer et al. 2005). Lichenan consists of repeating glucose units linked by β-1,3 and β-1,4 glycosidic bonds. The main product peaks detected are consistent with the masses of sodium adducts of dimeric to octameric β-d-Glc*p* (Fig. 4d). The specific activity of the enzyme towards xyloglucan and lichenan was 1.8- and 1.7-fold of that for CMC, respectively (Additional file 1: Table S2). This indicated that the recombinant GH 12 enzyme specifically recognized and hydrolyzed the β-1,4-glycosidic bond backbone of β-1,4-glucans. The enzyme showed high specific activity toward xyloglucan, suggesting that this enzyme was a XEG (EC 3.2.1.151). Thus, we named the GH12 enzyme as FsXEG12A.

### Biochemical characterization of recombinant FsXEG12A

**Fig. 5:**
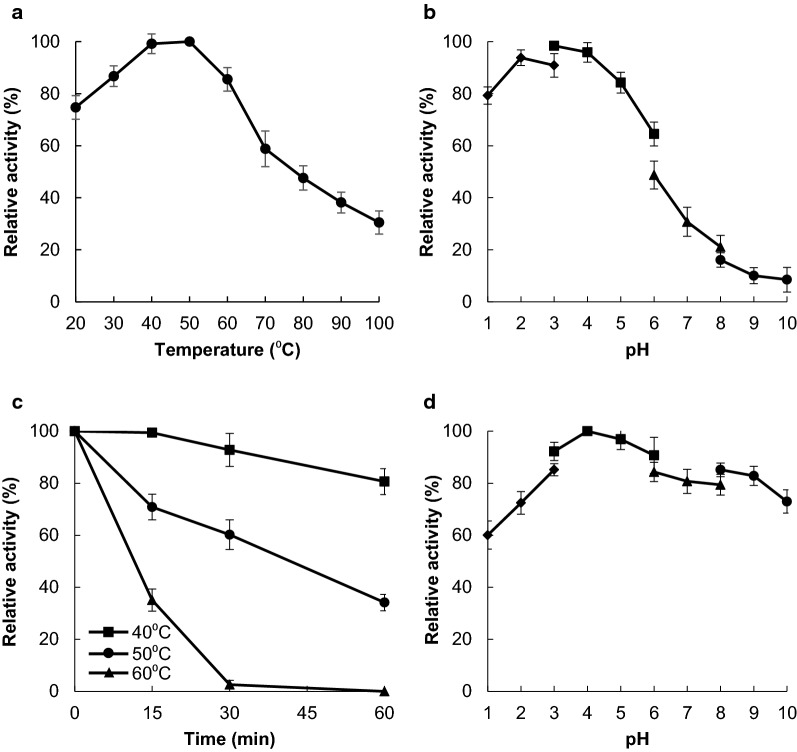
Biochemical characterization of FsXEG12A. **a** Optimal temperature of FsXEG12A. Enzyme reactions proceeded at temperatures ranging from 20 to 100 °C for 60 min. **b** Optimal pH of FsXEG12A. Enzyme reactions proceeded over a pH range of 1.0–10.0 in 50 mM glycine–HCl (pH 1.0–3.0, filled diamond), 50 mM sodium acetate (pH 3.0–6.0, filled square), 50 mM sodium phosphate (pH 6.0–8.0, filled triangle), and 50 mM Tris–HCl (pH 8.0–10.0, filled circle). **c** Thermostability of FsXEG12A. Purified FsXEG12A was incubated from 40 to 60 °C for 60 min, and then residual activity against 1.0% CMC was measured after further incubation at 50 °C for 60 min at the optimal pH (pH 3.0). **d** pH-stability of FsXEG12A. Purified FsXEG12A was incubated in 50 mM glycine–HCl (pH 1.0–3.0, filled diamond), 50 mM sodium acetate (pH 3.0–6.0, filled square), 50 mM sodium phosphate (pH 6.0–8.0, filled triangle) and 50 mM Tris–HCl (pH 8.0–10.0, filled circle) at 4 °C for 24 h. Residual enzyme activity against 1.0% CMC was then measured at the optimal pH (pH 3.0). Data are presented as mean ± standard deviation of three experiments

The optimal temperature and pH for FsXEG12A were determined using CMC. The optimal reaction temperature range for FsXEG12A was 20–60 °C (Fig. 5a), whereas the optimal pH was 3.0, with a preferred pH range between 1.0 and 5.0 (Fig. 5b). We determined the kinetic parameters of FsXEG12A using CMC, MCC, xyloglucan, lichenan and glucomannan (Table 3). The catalytic efficiency (*k*_cat_/*K*_m_) of FsXEG12A was higher toward xyloglucan (1027.0 mL/s/mg) and lichenan (845.2 mL/s/mg) than toward CMC (65.5 mL/s/mg).

**Table 3:** Apparent kinetic parameters of the purified FsXEG12A toward the various polysaccharides

Substrate	*K*_*m*_ (mg/mL)	*k*_cat_ (/s)	*k*_cat_/*K*_*m*_ (mL/s/mg)
CMC	5.8 ± 0.4	380 ± 20	65.5
MCC	34.7 ± 5.7	55 ± 2	1.6
Xyloglucan	0.74 ± 0.06	760 ± 30	1027.0
Lichenan	0.84 ± 0.04	710 ± 20	845.2
Glucomannan	7.8 ± 1.1	350 ± 40	44.9

Data are mean ± standard deviation of three experiments. Recombinant FsXEG12A (1.0 μM) was incubated with substrates in 50 mM acetate buffer (pH 3.0) at 37 °C

The thermostability and pH stability of FsXEG12A were investigated using CMC as the substrate. Purified FsXEG12A was incubated at 40 °C, 50 °C, or 60 °C for 60 min (Fig. 5c). After incubation at 40 °C for 60 min, the residual activity of FsXEG12A decreased to 81% of its maximal activity. After incubation at 50 °C for 30 min, the residual activity decreased to 60%. After incubation for 24 h at 4 °C at pH ranging from 1.0 to 10.0, FsXEG12A retained > 60% of its maximal activity (Fig. 5d). These findings indicated that FsXEG12A remains highly stable over a pH range of 1.0 to 10.0.

## Discussion

A microbial analysis of *16S rRNA* and *18S rRNA* genes extracted directly from the microbial community in tunnels excavated by *E. interjectus* was performed using the DGGE method. Seven gram-positive bacteria, seven gram-negative bacteria, and one fungus were identified (Table 2). Previous studies have shown that *E. interjectus* is a vector of *Ceratocystis ficicola* (Kajitani 1996; Morita et al. 2012) and that a wide distribution of *C. ficicola* in sapwood causes wilt symptoms in trees (Kajii et al. 2013). However, in this study, DGGE analysis identified only *Fusarium* sp. as a filamentous fungus (Fig. 2a). *Fusarium* spp. strain EI was isolated from the microorganisms obtained from the excavated tunnels and from *E. interjectus*. Prospective plant pathogens must overcome the physical barrier presented by the plant cell wall. Plant pathogenic fungi are known for their ability to degrade and assimilate wood components (e.g. cellulose and hemicellulose) and to penetrate the plant cell (Underwood 2012). Our findings identified the *E. interjectus*-cultured *Fusarium* spp. strain EI as a cellulose-degrading fungus that produces the XEG FsXEG12A.

Xyloglucan consists of β-1,4-glucan with xylosyl side chains attached to the O-6 position of glycosyl residues, and it associates with cellulose microfibrils through hydrogen bonds, forming a cellulose–xyloglucan network (Damásio et al. 2017; Pauly and Keegstra 2016; Yang et al. 2017). Xyloglucanases (XEGs) are responsible for the hydrolysis of the xyloglucan backbone. Generally, XEG activities are significant towards xyloglucan; however, many XEGs belonging to GH12 and GH74 have low or no activity towards CMC, MCC, lichenan, glucomannan, and xylan (Desmet et al. 2007; Gloster et al. 2007; Sato et al. 2016; Takeda et al. 2013). Highly xyloglucan-specific GH12 XEGs have been isolated from various fungi including *Aspergillus niveus* (Damásio et al. 2012), *Aspergillus terreus* (Vitcosque et al. 2016), *Aspergillus niger* (Master et al. 2008), and *Aspergillus cervinus* (Rykov et al. 2019). Unlike xyloglucan-specific GH12 XEGs, some fungal GH12 XEGs with broad substrate specificity have also been reported (Segato et al. 2017; Yang et al. 2017). When the specific activity of FsXEG12A towards xyloglucan is set to 100%, its activities towards CMC, MCC, lichenan, and glucomannan are 59%, 21%, 98%, and 45%, respectively (Additional file 1: Table S2). These results indicate that the specific activities of FsXEG12A towards the substrates are at least equal to those of other GH12 XEGs with broad substrate specificity (Segato et al. 2017; Yang et al. 2017). FsXEG12A degrades both xyloglucan and cellulose, resulting in weakening of the cellulose–xyloglucan network. This allows the fungus to infect the plants and utilize the reaction products of cellulose and xyloglucan for fungal growth. Therefore, the broad substrate specificity of FsXEG12A from *Fusarium* spp. strain EI is beneficial for degrading the complex wood components such as cellulose, xyloglucan, galactoglucomannan, and xylan, which compose 30–40%, 20–25%, 4–8%, and 12–18%, respectively, of the mass of angiosperm plants (Rytioja et al. 2014).

The virulence factors secreted from *F. oxysporum* have been found to function at different stages of the infection process to induce disease and counteract the plant defence reactions (Roncero et al. 2003). *F. oxysporum* can produce several toxins such as fusaric acid, beauvericin, enniatin B, bikaverin, moniliformin, fumonisin, and trichothecenes (Irzykowska et al. 2012; Mirocha et al. 1989; Moretti et al. 2002; Son et al. 2008). The potential roles of these toxins in the pathogenic process are not fully understood. Recently, Ma et al. (2015) reported that XEG1 belonging to GH12 not only acts as an XEG but also works as a pathogen-associated molecular pattern (PAMP) in angiosperm plants (for example, soybean and solanaceous species) and that XEG1 triggers cell death (Ma et al. 2015). Transcriptional responses involved in PAMP have been shown to occur in apples, which belong to the order Rosales, as are figs (Puławska et al. 2017). This suggests that the production of GH12 proteins, including FsXEG12, triggers cell death in plants, thereby facilitating infection. FsXEG12 produced by *Fusarium* spp. strain EI degrades wood components and is critical as a potential virulence factor. Infection with *Fusarium* spp. could, thus, result in progressive wilting, which often occurs by the blockage of dead vessels through mycelial growth leading to the death of the plant.

For many plant pathogens, including *Fusarium* species, fungicides have been applied for disease management of the host. Some benzoic acid analogues such as gallic acid, ferulic acid, *p*-hydroxybenzoic acid, and cuminic acid strongly inhibit *F. oxysporum* spp. growth (Sun et al. 2017; Wu et al. 2009, 2010a, b), suggesting that these biofungicides could be used for inhibiting the *Fusarium* dieback mediated by *Euwallacea* spp. It has also been reported that vanillin, syringaldehyde, *trans*-cinnamic acid, and hydroxybenzoic acid inhibit cellulose hydrolysis in wet cake (Qin et al. 2016). Therefore, benzoic acid analogues could be used as lead compounds for the design of inhibitors against the cellulase secreted by *Fusarium* species.

In this study, the cellulose-degrading fungus *Fusarium* spp. strain EI was isolated from the microbial community cultivated by *E. interjectus*. Our biochemical characterization of the FsXEG12A enzyme produced by the fungus suggests that the broad substrate specificity of FsXEG12A facilitates the broad species specificity of *E. interjectus*. Inhibition of FsXEG12A function is, thus, an effective target for reducing *Fusarium* dieback caused by *Euwallacea* spp.

## Supplementary information

**Additional file 1: Table S1.** Identification of the cellulolytic enzyme produced by *Fusarium* spp. strain EI. **Table S2.** Substrate specificity of the purified GH12 enzyme. **Figure S1.** Nucleotide sequence alignment of *18S rRNA*. **Figure S2.** Nucleotide sequence alignment of *EF-1α*. **Figure S3.** Amino acid sequence alignment of GH12 enzymes.

## Data Availability

Corresponding author could provide all the experimental data on reasonable request.
