# Supplementary material for: Characterization of FsXEG12A from the cellulose-degrading ectosymbiotic fungus Fusarium spp. strain EI cultured by the ambrosia beetle
Source: AMB Express. 2020 May 24;10:96. doi: 10.1186/s13568-020-01030-6 (PMC7246284; doi:10.1186/s13568-020-01030-6)

AMB Express

Supplemental information for

Characterization of FsXEG12A from the cellulose-degrading ectosymbiotic fungus *Fusarium* spp. strain EI cultured by the ambrosia beetle

Kiyota Sakai[1], Aya Yamaguchi[1], Seitaro Tsutsumi[1], Yuto Kawai[1], Sho Tsuzuki[1], Hiromitsu Suzuki[1], Sadanari Jindou[2], Yoshihito Suzuki[3], Hisashi Kajimura[3], Masashi Kato[1], Motoyuki Shimizu[1]*

[1] *Faculty of Agriculture, Meijo University, Nagoya, Aichi, 468-8502, Japan*

[2] *Faculty of Science and Technology, Meijo University, Nagoya, Aichi, 468-8502, Japan*

[3] *Graduate School of Bioagricultural Sciences, Nagoya University, Nagoya, Aichi, 464-8601, Japan*

*****Corresponding author. Tel/Fax: +81-52-838-2445

E-mail: moshimi@meijo-u.ac.jp (M. Shimizu)

Table S1. Identification of the cellulolytic enzyme produced by *Fusarium* spp. strain EI

Annotation name	Gene ID[a]	PMF search			MS/MS ion search
		Score	MW[b]	Cov.[c]	Identified peptide sequence
Hypothetical protein	EMT67806.1	127	20.9	54	GSYTVSGLGQR, TYDAANFGLFK

[a]Protein name and accession numbers were obtained from the NCBI protein database.

[b]Theoretical mass.

[c]Sequence coverage (%) in peptide mass fingerprinting (PMF).

Table S2. Substrate specificity of the purified GH12 enzyme

Substrate	Specific activity (U/mL)	Relative activity (%)
CMC	158.9 ± 10.7	59.2
MCC	57.3 ± 9.4	21.3
Xyloglucan	268.6 ± 50.1	100
Lichenan	263.7 ± 37.5	98.2
Glucomannan	121.3 ± 14.4	45.2

Data are means ± standard deviation of three experiments. Recombinant GH12 enzyme (1.0 μM) was incubated with substrates in 50 mM acetate buffer (pH 3.0) at 37°C.

Figure S1. Nucleotide sequence alignment of *18S rRNA*.

18S rRNA sequences of *Fusarium* spp. were aligned using ClustalW. Identical nucleotides are highlighted. Dashes indicate gaps. *18S rRNA* sequences of LC534254 from *Fusarium* spp. strain EI, LT841222.1 from *F. oxysporum* f. sp. *dianthi* strain Fod001, LT841236.1 from *F. oxysporum* f. sp. *dianthi* strain Fod008, LT841208.1 from *F. oxysporum* f. sp. *cumini* strain F11, LT841250.1 from *F. proliferatum* strain ITEM2287, LT841264.1 from *F. proliferatum* strain ITEM2400, KC866343.1 from *F. sporotrichioides* isolate CHILEFUS1, KC866344.1 from *F. sporotrichioides* isolate CHILEFUS2, KP310124.1 from *F. solani* strain XXG-2, EF397944.1 from *F. solani* strain 421502, KX132128.1 from *F. graminearum* strain CBE330.6, and KX132126.1 from *F. graminearum* strain CBE330.5 are shown.

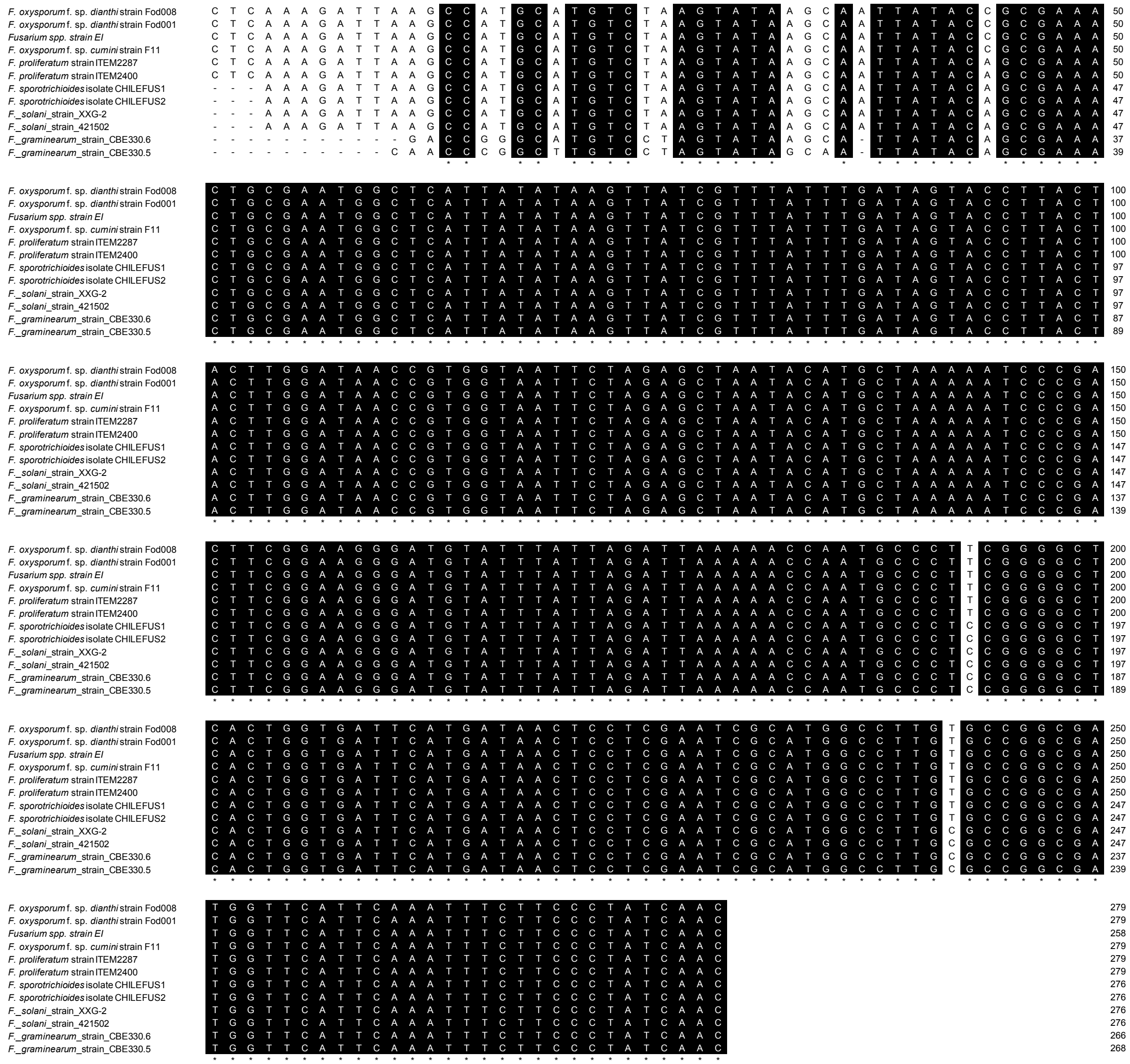
F. oxysporum f. sp. dianthi strain Fod008 CTCAAAGATTAAGCCATGCATGTCTAAGTATAAGCAATTATACCGCGAAA 50
F. oxysporum f. sp. dianthi strain Fod001 CTCAAAGATTAAGCCATGCATGTCTAAGTATAAGCAATTATACCGCGAAA 50
Fusarium spp. strain EI CTCAAAGATTAAGCCATGCATGTCTAAGTATAAGCAATTATACCGCGAAA 50
F. oxysporum f. sp. cumini strain F11 CTCAAAGATTAAGCCATGCATGTCTAAGTATAAGCAATTATACCGCGAAA 50
F. proliferatum strain ITEM2287 CTCAAAGATTAAGCCATGCATGTCTAAGTATAAGCAATTATACAGCGAAA 50
F. proliferatum strain ITEM2400 CTCAAAGATTAAGCCATGCATGTCTAAGTATAAGCAATTATACAGCGAAA 50
F. sporotrichioides isolate CHILEFUS1 ---AAAGATTAAGCCATGCATGTCTAAGTATAAGCAATTATACAGCGAAA 47
F. sporotrichioides isolate CHILEFUS2 ---AAAGATTAAGCCATGCATGTCTAAGTATAAGCAATTATACAGCGAAA 47
F._solani_strain_XXG-2 ---AAAGATTAAGCCATGCATGTCTAAGTATAAGCAATTATACAGCGAAA 47
F._solani_strain_421502 ---AAAGATTAAGCCATGCATGTCTAAGTATAAGCAATTATACAGCGAAA 47
F._graminearum_strain_CBE330.6 -----------GACCGGGCATGTCCTAGTATAAGCA-TTATACAGCGAAA 37
F._graminearum_strain_CBE330.5 ----------CAACCCGGCTTGTCCTAGTATAGCAA-TTATACAGCGAAA 39
F. oxysporum f. sp. dianthi strain Fod008 CTGCGAATGGCTCATTATATAAGTTATCGTTTATTTGATAGTACCTTACT 100
F. oxysporum f. sp. dianthi strain Fod001 CTGCGAATGGCTCATTATATAAGTTATCGTTTATTTGATAGTACCTTACT 100
Fusarium spp. strain EI CTGCGAATGGCTCATTATATAAGTTATCGTTTATTTGATAGTACCTTACT 100
F. oxysporum f. sp. cumini strain F11 CTGCGAATGGCTCATTATATAAGTTATCGTTTATTTGATAGTACCTTACT 100
F. proliferatum strain ITEM2287 CTGCGAATGGCTCATTATATAAGTTATCGTTTATTTGATAGTACCTTACT 100
F. proliferatum strain ITEM2400 CTGCGAATGGCTCATTATATAAGTTATCGTTTATTTGATAGTACCTTACT 100
F. sporotrichioides isolate CHILEFUS1 CTGCGAATGGCTCATTATATAAGTTATCGTTTATTTGATAGTACCTTACT 97
F. sporotrichioides isolate CHILEFUS2 CTGCGAATGGCTCATTATATAAGTTATCGTTTATTTGATAGTACCTTACT 97
F._solani_strain_XXG-2 CTGCGAATGGCTCATTATATAAGTTATCGTTTATTTGATAGTACCTTACT 97
F._solani_strain_421502 CTGCGAATGGCTCATTATATAAGTTATCGTTTATTTGATAGTACCTTACT 97
F._graminearum_strain_CBE330.6 CTGCGAATGGCTCATTATATAAGTTATCGTTTATTTGATAGTACCTTACT 87
F._graminearum_strain_CBE330.5 CTGCGAATGGCTCATTATATAAGTTATCGTTTATTTGATAGTACCTTACT 89
F. oxysporum f. sp. dianthi strain Fod008 ACTTGGATAACCGTGGTAATTCTAGAGCTAATACATGCTAAAAATCCCGA 150
F. oxysporum f. sp. dianthi strain Fod001 ACTTGGATAACCGTGGTAATTCTAGAGCTAATACATGCTAAAAATCCCGA 150
Fusarium spp. strain EI ACTTGGATAACCGTGGTAATTCTAGAGCTAATACATGCTAAAAATCCCGA 150
F. oxysporum f. sp. cumini strain F11 ACTTGGATAACCGTGGTAATTCTAGAGCTAATACATGCTAAAAATCCCGA 150
F. proliferatum strain ITEM2287 ACTTGGATAACCGTGGTAATTCTAGAGCTAATACATGCTAAAAATCCCGA 150
F. proliferatum strain ITEM2400 ACTTGGATAACCGTGGTAATTCTAGAGCTAATACATGCTAAAAATCCCGA 150
F. sporotrichioides isolate CHILEFUS1 ACTTGGATAACCGTGGTAATTCTAGAGCTAATACATGCTAAAAATCCCGA 147
F. sporotrichioides isolate CHILEFUS2 ACTTGGATAACCGTGGTAATTCTAGAGCTAATACATGCTAAAAATCCCGA 147
F._solani_strain_XXG-2 ACTTGGATAACCGTGGTAATTCTAGAGCTAATACATGCTAAAAATCCCGA 147
F._solani_strain_421502 ACTTGGATAACCGTGGTAATTCTAGAGCTAATACATGCTAAAAATCCCGA 147
F._graminearum_strain_CBE330.6 ACTTGGATAACCGTGGTAATTCTAGAGCTAATACATGCTAAAAATCCCGA 137
F._graminearum_strain_CBE330.5 ACTTGGATAACCGTGGTAATTCTAGAGCTAATACATGCTAAAAATCCCGA 139
F. oxysporum f. sp. dianthi strain Fod008 CTTCGGAAGGGATGTATTTATTAGATTAAAAACCAATGCCCTTCGGGGCT 200
F. oxysporum f. sp. dianthi strain Fod001 CTTCGGAAGGGATGTATTTATTAGATTAAAAACCAATGCCCTTCGGGGCT 200
Fusarium spp. strain EI CTTCGGAAGGGATGTATTTATTAGATTAAAAACCAATGCCCTTCGGGGCT 200
F. oxysporum f. sp. cumini strain F11 CTTCGGAAGGGATGTATTTATTAGATTAAAAACCAATGCCCTTCGGGGCT 200
F. proliferatum strain ITEM2287 CTTCGGAAGGGATGTATTTATTAGATTAAAAACCAATGCCCTTCGGGGCT 200
F. proliferatum strain ITEM2400 CTTCGGAAGGGATGTATTTATTAGATTAAAAACCAATGCCCTTCGGGGCT 200
F. sporotrichioides isolate CHILEFUS1 CTTCGGAAGGGATGTATTTATTAGATTAAAAACCAATGCCCTCCGGGGCT 197
F. sporotrichioides isolate CHILEFUS2 CTTCGGAAGGGATGTATTTATTAGATTAAAAACCAATGCCCTCCGGGGCT 197
F._solani_strain_XXG-2 CTTCGGAAGGGATGTATTTATTAGATTAAAAACCAATGCCCTCCGGGGCT 197
F._solani_strain_421502 CTTCGGAAGGGATGTATTTATTAGATTAAAAACCAATGCCCTCCGGGGCT 197
F._graminearum_strain_CBE330.6 CTTCGGAAGGGATGTATTTATTAGATTAAAAACCAATGCCCTCCGGGGCT 187
F._graminearum_strain_CBE330.5 CTTCGGAAGGGATGTATTTATTAGATTAAAAACCAATGCCCTCCGGGGCT 189
F. oxysporum f. sp. dianthi strain Fod008 CACTGGTGATTCATGATAACTCCTCGAATCGCATGGCCTTGTGCCGGCGA 250
F. oxysporum f. sp. dianthi strain Fod001 CACTGGTGATTCATGATAACTCCTCGAATCGCATGGCCTTGTGCCGGCGA 250
Fusarium spp. strain EI CACTGGTGATTCATGATAACTCCTCGAATCGCATGGCCTTGTGCCGGCGA 250
F. oxysporum f. sp. cumini strain F11 CACTGGTGATTCATGATAACTCCTCGAATCGCATGGCCTTGTGCCGGCGA 250
F. proliferatum strain ITEM2287 CACTGGTGATTCATGATAACTCCTCGAATCGCATGGCCTTGTGCCGGCGA 250
F. proliferatum strain ITEM2400 CACTGGTGATTCATGATAACTCCTCGAATCGCATGGCCTTGTGCCGGCGA 250
F. sporotrichioides isolate CHILEFUS1 CACTGGTGATTCATGATAACTCCTCGAATCGCATGGCCTTGTGCCGGCGA 247
F. sporotrichioides isolate CHILEFUS2 CACTGGTGATTCATGATAACTCCTCGAATCGCATGGCCTTGTGCCGGCGA 247
F._solani_strain_XXG-2 CACTGGTGATTCATGATAACTCCTCGAATCGCATGGCCTTGCGCCGGCGA 247
F._solani_strain_421502 CACTGGTGATTCATGATAACTCCTCGAATCGCATGGCCTTGCGCCGGCGA 247
F._graminearum_strain_CBE330.6 CACTGGTGATTCATGATAACTCCTCGAATCGCATGGCCTTGCGCCGGCGA 237
F._graminearum_strain_CBE330.5 CACTGGTGATTCATGATAACTCCTCGAATCGCATGGCCTTGCGCCGGCGA 239
F. oxysporum f. sp. dianthi strain Fod008 TGGTTCATTCAAATTTCTTCCCTATCAAC 279
F. oxysporum f. sp. dianthi strain Fod001 TGGTTCATTCAAATTTCTTCCCTATCAAC 279
Fusarium spp. strain EI TGGTTCATTCAAATTTCTTCCCTATCAAC 258
F. oxysporum f. sp. cumini strain F11 TGGTTCATTCAAATTTCTTCCCTATCAAC 279
F. proliferatum strain ITEM2287 TGGTTCATTCAAATTTCTTCCCTATCAAC 279
F. proliferatum strain ITEM2400 TGGTTCATTCAAATTTCTTCCCTATCAAC 279
F. sporotrichioides isolate CHILEFUS1 TGGTTCATTCAAATTTCTTCCCTATCAAC 276
F. sporotrichioides isolate CHILEFUS2 TGGTTCATTCAAATTTCTTCCCTATCAAC 276
F._solani_strain_XXG-2 TGGTTCATTCAAATTTCTTCCCTATCAAC 276
F._solani_strain_421502 TGGTTCATTCAAATTTCTTCCCTATCAAC 276
F._graminearum_strain_CBE330.6 TGGTTCATTCAAATTTCTTCCCTATCAAC 266
F._graminearum_strain_CBE330.5 TGGTTCATTCAAATTTCTTCCCTATCAAC 268

Figure S2. Nucleotide sequence alignment of *EF-1α*.

The nucleotide sequence alignment was performed using partial *EF-1α* sequences of *Fusarium* spp. strain EI, *Fusarium* species registered in the JGI database (https://mycocosm.jgi.doe.gov/Fusarium/Fusarium.info.html) and the *Fusarium* sp. AF-3 sequence. The *EF-1α* sequences of *Fusarium* spp. were aligned using ClustalW. Identical nucleotides are highlighted. Dashes indicate gaps. *EF-1α* sequences of KC691533.1 from *Fusarium* sp. AF-3, KC691536.1 from *Fusarium* sp. AF-3, 599354 from *F. commune* MPI-SDFR-AT-0072, 8475 from *F. fujikuroi* IMI 58289, 9400 from *F. graminearum* FGSG_08811T0, 17733 from *F. oxysporum* f. sp. *lycopersici* 4287, 554906 from *F. oxysporum* MPI-SDFR-AT-0094, 4767 from *F. pseudograminearum* CS3096, 485664 from *F. solani* FSSC 5, 10379 from *F. verticillioides* 7600, and LC534255 from *Fusarium* spp. strain EI are shown.

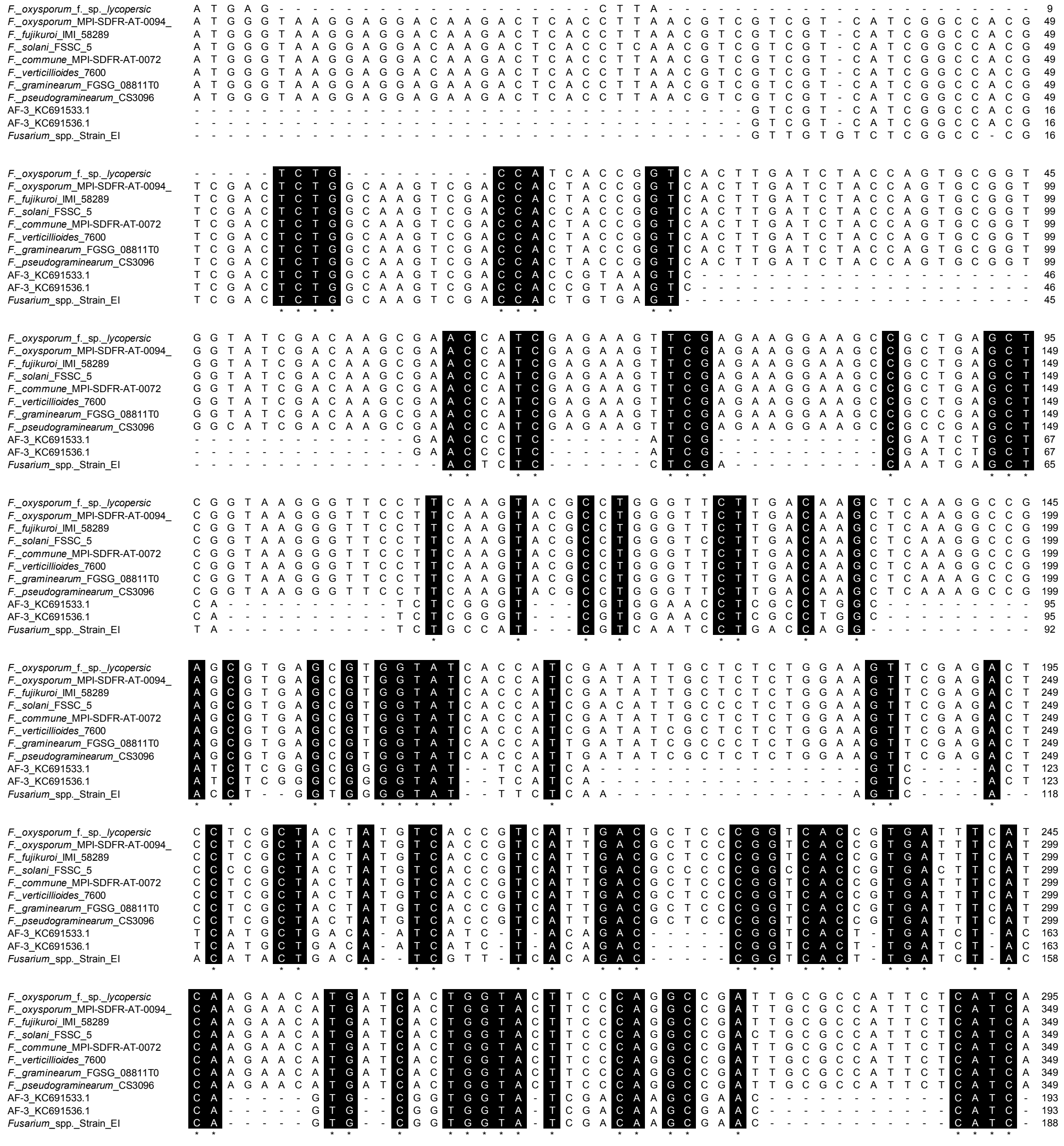

```
F._oxysporum_f._sp._lycopersic  TTGCCGCCGGTACTGGTGAGTTCGAGGCTGGTATCTCCAAGGATGGCCAG 345
F._oxysporum_MPI-SDFR-AT-0094_  TTGCCGCCGGTACTGGTGAGTTCGAGGCTGGTATCTCCAAGGATGGCCAG 399
F._fujikuroi_IMI_58289          TTGCCGCCGGTACTGGTGAGTTCGAGGCTGGTATCTCCAAGGATGGCCAG 399
F._solani_FSSC_5                TTGCCGCCGGTACTGGTGAGTTCGAGGCTGGTATCTCCAAGGATGGCCAG 399
F._commune_MPI-SDFR-AT-0072     TTGCCGCCGGTACTGGTGAGTTCGAGGCTGGTATCTCCAAGGATGGCCAG 399
F._verticillioides_7600         TTGCCGCCGGTACTGGTGAGTTCGAGGCTGGTATCTCCAAGGATGGCCAG 399
F._graminearum_FGSG_08811T0     TTGCCGCCGGTACTGGTGAGTTCGAGGCTGGTATCTCCAAGGATGGCCAG 399
F._pseudograminearum_CS3096     TTGCCGCCGGTACTGGTGAGTTCGAGGCTGGTATCTCCAAGGATGGCCAG 399
AF-3_KC691533.1                 --------------GAGAAGTTCGAG------------AAGGTTGGTGA- 216
AF-3_KC691536.1                 --------------GAGAAGTTCGAG------------AAGGTTGGTGA- 216
Fusarium_spp._Strain_EI         --------------GAGAAGTTCGAG------------AAGGTTAGTCA- 211
                                              *   ********            **** * *  *

F._oxysporum_f._sp._lycopersic  ACCCGTGAGCACGCTCTTCTTGCCTACACCCTTGGTGTCAAGAACCTCAT 395
F._oxysporum_MPI-SDFR-AT-0094_  ACCCGTGAGCACGCTCTTCTTGCCTACACCCTTGGTGTCAAGAACCTCAT 449
F._fujikuroi_IMI_58289          ACCCGTGAGCACGCTCTTCTTGCCTACACCCTTGGTGTCAAGAACCTCAT 449
F._solani_FSSC_5                ACCCGTGAGCACGCCCTGCTCGCCTACACCCTCGGTGTCAAGAACCTCAT 449
F._commune_MPI-SDFR-AT-0072     ACCCGTGAGCACGCTCTTCTTGCCTACACCCTTGGTGTCAAGAACCTCAT 449
F._verticillioides_7600         ACCCGTGAGCACGCTCTTCTTGCCTACACCCTCGGTGTCAAGAACCTCAT 449
F._graminearum_FGSG_08811T0     ACCCGTGAGCACGCTCTCCTTGCCTACACCCTTGGTGTCAAGAACCTCAT 449
F._pseudograminearum_CS3096     ACCCGTGAGCACGCTCTTCTTGCCTACACTCTTGGTGTCAAGAACCTCAT 449
AF-3_KC691533.1                 ---------------CATCT--------CCCCCG--------------AT 229
AF-3_KC691536.1                 ---------------CATCT--------CCCCCG--------------AT 229
Fusarium_spp._Strain_EI         ---------------CTTTC--------CCTTCG--------------AT 224
                                               *            *    *              **

F._oxysporum_f._sp._lycopersic  TGTCGCCATCAACAAGATGGACACCACCAAGTGGTCTGAGGCCCGTTACC 445
F._oxysporum_MPI-SDFR-AT-0094_  TGTCGCCATCAACAAGATGGACACCACCAAGTGGTCTGAGGCCCGTTACC 499
F._fujikuroi_IMI_58289          CGTCGCCATCAACAAGATGGACACCACCAAGTGGTCTGAGGCCCGTTACC 499
F._solani_FSSC_5                TGTCGCCATCAACAAGATGGACACCACCAAGTGGTCCGAGTCCCGTTACC 499
F._commune_MPI-SDFR-AT-0072     CGTCGCCATCAACAAGATGGACACCACCAAGTGGTCTGAGGCCCGTTACC 499
F._verticillioides_7600         CGTCGCCATCAACAAGATGGACACCACCAAGTGGTCTGAGGCCCGTTACC 499
F._graminearum_FGSG_08811T0     TGTTGCCATCAACAAGATGGACACCACCAAGTGGTCTGAGGCCCGTTACC 499
F._pseudograminearum_CS3096     TGTTGCCATCAACAAGATGGACACCACCAAGTGGTCTGAGGCCCGTTACC 499
AF-3_KC691533.1                 CG-CGCCTT-------------GCTATC-------CCACATCGAATTCCC 258
AF-3_KC691536.1                 CG-CGCCTT-------------GCTATC-------CCACATCGAATTCCC 258
Fusarium_spp._Strain_EI         CG-CGC-------------------GTC-------CT--------TTGCC 239
                                 *  **                     *       *         ** **

F._oxysporum_f._sp._lycopersic  AGGAGATCATCAAGGAGACCTCCTCTTTCATCAAGAAGGTCGGCTACAAC 495
F._oxysporum_MPI-SDFR-AT-0094_  AGGAGATCATCAAGGAGACCTCCTCTTTCATCAAGAAGGTCGGCTACAAC 549
F._fujikuroi_IMI_58289          AGGAGATCATCAAGGAGACCTCCTCTTTCATCAAGAAGGTCGGCTACAAC 549
F._solani_FSSC_5                AGGAGATCATCAAGGAGACCTCCAACTTTCATCAAGAAGGTCGGCTACAAC 549
F._commune_MPI-SDFR-AT-0072     AGGAGATCATCAAGGAGACTTCTTCTTTCATCAAGAAGGTCGGCTACAAC 549
F._verticillioides_7600         AGGAAATCATCAAGGAGACCTCCTCTTTCATCAAGAAGGTCGGCTACAAC 549
F._graminearum_FGSG_08811T0     AGGAGATCATCAAGGAGACCTCTTCTTTCATCAAGAAGGTCGGCTACAAC 549
F._pseudograminearum_CS3096     AGGAGATCATCAAGGAGACCTCCTCTTTCATCAAGAAGGTCGGCTACAAC 549
AF-3_KC691533.1                 -------CGTC-----GAATTCCCTCCC---------------TCACGAT 281
AF-3_KC691536.1                 -------CGTC-----GAATTCCCTCCC---------------TCACGAT 281
Fusarium_spp._Strain_EI         -------CATC-----GATTTCCC-------------------CTACGAC 258
                                       * **     **  **                       ** *

F._oxysporum_f._sp._lycopersic  CCCAAGGCTGTCGCTTTCGTCCCCATCTCCGGTTTCAACGGTGACAACAT 545
F._oxysporum_MPI-SDFR-AT-0094_  CCCAAGGCTGTCGCTTTCGTCCCCATCTCCGGTTTCAACGGTGACAACAT 599
F._fujikuroi_IMI_58289          CCCAAGGCTGTCGCTTTCGTCCCCATCTCCGGTTTCAACGGTGACAACAT 599
F._solani_FSSC_5                CCCAAGGCTGTCGCTTTCGTCCCCATCTCCGGTTTCAACGGCGACAACAT 599
F._commune_MPI-SDFR-AT-0072     CCCAAGGCTGTCGCTTTCGTCCCCATCTCCGGTTTCAACGGTGACAACAT 599
F._verticillioides_7600         CCCAAGGCTGTCGCTTTCGTCCCCATCTCCGGCTTCAACGGTGACAACAT 599
F._graminearum_FGSG_08811T0     CCCAAGGCTGTCGCTTTCGTCCCCATCTCCGGTTTCAACGGTGACAACAT 599
F._pseudograminearum_CS3096     CCCAAGGCTGTCGCTTTCGTCCCCATCTCCGGTTTCAACGGTGACAACAT 599
AF-3_KC691533.1                 CC-----------------------------------------GCGCTGC 290
AF-3_KC691536.1                 CC-----------------------------------------GCGCTGC 290
Fusarium_spp._Strain_EI         TC-----------------------------------------GAAACGT 267
                                 *

F._oxysporum_f._sp._lycopersic  GCTTACCCCCTCCACCAACTGCCCCTGGTACAAGGGTTGGGAGCGTGAGA 595
F._oxysporum_MPI-SDFR-AT-0094_  GCTTACCCCCTCCACCAACTGCCCCTGGTACAAGGGTTGGGAGCGTGAGA 649
F._fujikuroi_IMI_58289          GCTTACCCCCTCCACCAACTGCCCCTGGTACAAGGGTTGGGAGCGTGAGA 649
F._solani_FSSC_5                GCTTACTCCCTCCACCAACTGCCCCTGGTACAAGGGCTGGGAGCGTGAGA 649
F._commune_MPI-SDFR-AT-0072     GCTTACCCCCTCCACCAACTGCCCCTGGTACAAGGGTTGGGAGCGTGAGA 649
F._verticillioides_7600         GCTTACCCCCTCCACCAACTGCCCTTGGTACAAGGGTTGGGAGCGTGAGA 649
F._graminearum_FGSG_08811T0     GCTTACTGCCTCCACCAACTGCCCCTGGTACAAGGGTTGGGAGCGTGAGA 649
F._pseudograminearum_CS3096     GCTTGCCCCCTCCACCAACTGCCCCTGGTACAAGGGTTGGGAGCGTGAGA 649
AF-3_KC691533.1                 GCCCGCTTCTCC---CGA--GTCC-----CAAAATTTTTGCGGTGCGA-- 328
AF-3_KC691536.1                 GCCCGCTTCTCC---CGA--GTCC-----CAAAATTTTTGCGGTGCGA-- 328
Fusarium_spp._Strain_EI         ACCCGCTACCCCGCTCGA--GACC-----AAAAATTTT-GCAATATGA-- 307
                                 *   *  *  *   * *  * **       **    * *      **
```

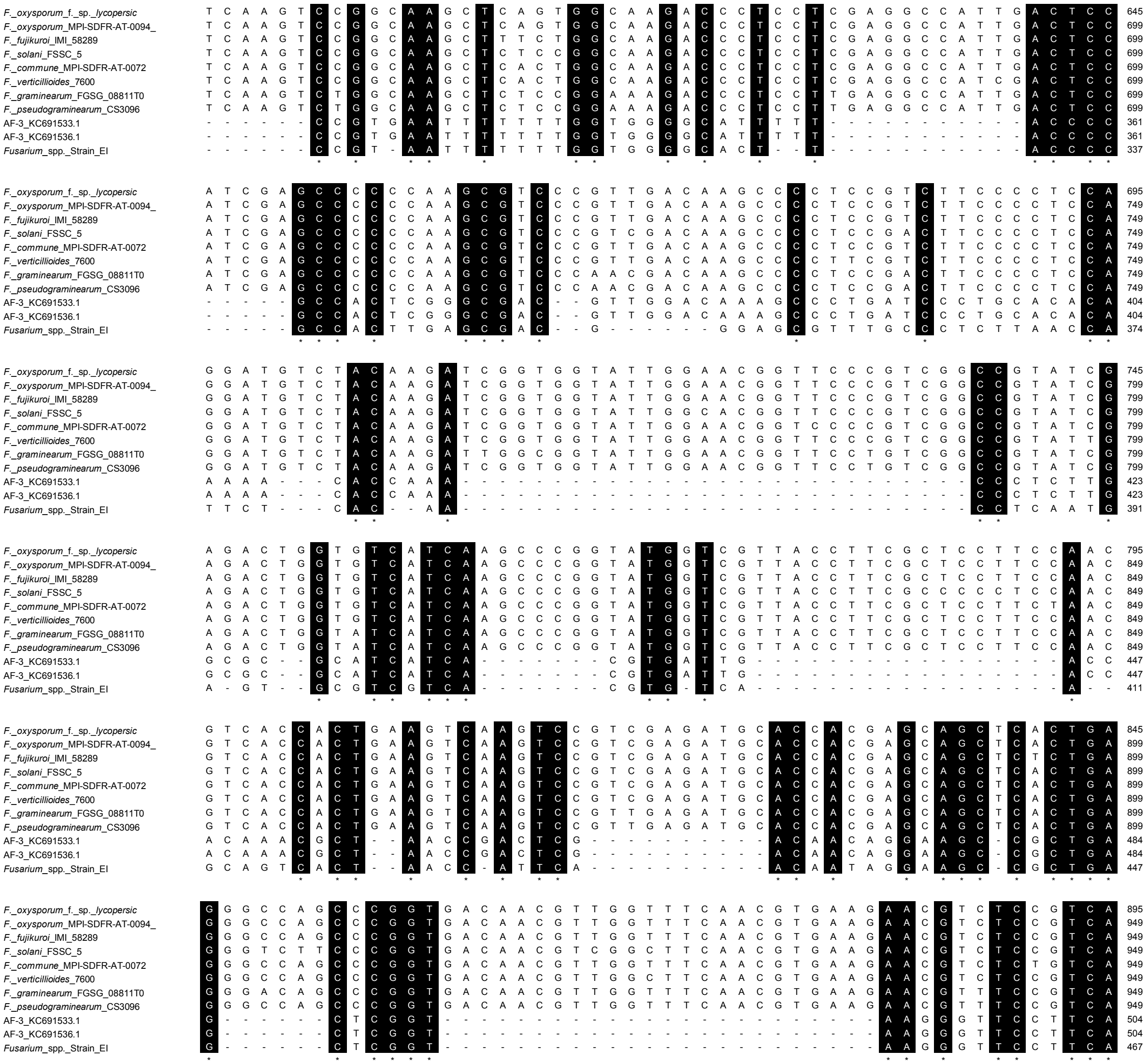

```
F._oxysporum_f._sp._lycopersic        AGGACATCCGACGTGGTAACGTCGCTGGTGACTCCAAGAACGACCCCCCT  945
F._oxysporum_MPI-SDFR-AT-0094_        AGGACATCCGACGTGGTAACGTCGCTGGTGACTCCAAGAACGACCCCCCT  999
F._fujikuroi_IMI_58289                AGGACATCCGACGTGGTAACGTCGCTGGTGACTCCAAGAACGACCCCCCC  999
F._solani_FSSC_5                      AGGAGATCCGACGTGGCAACGTCGCTGGTGACTCCAAGAACGACCCCCCT  999
F._commune_MPI-SDFR-AT-0072           AGGACATCCGACGTGGTAACGTCGCTGGTGACTCCAAGAACGACCCCCCT  999
F._verticillioides_7600               AGGACATCCGACGTGGTAACGTCGCTGGTGACTCCAAGAACGACCCCCCC  999
F._graminearum_FGSG_08811T0           AGGACATCCGACGTGGTAACGTCGCTGGTGACTCCAAGAACGACCCCCCC  999
F._pseudograminearum_CS3096           AGGACATCCGACGTGGTAACGTCGCTGGTGACTCCAAGAACGACCCCCCC  999
AF-3_KC691533.1                       AGTACGCCTGG---------GTCCTTGACAAGCTCAAGGCCGAGC-----  540
AF-3_KC691536.1                       AGTACGCCTGG---------GTCCTTGACAAGCTCAAGGCCGAGC-----  540
Fusarium_spp._Strain_EI               AGTACGCCTGG---------GTTCTTGACAAGCTCAAGGCCGAGC-----  503
                                      ** *   * *          **   **   *    ****  *** *

F._oxysporum_f._sp._lycopersic        ATGGGTGCCGCTTCTTTCACCGCTCAGGTCATCGTCCTCAACCACCCCGG  995
F._oxysporum_MPI-SDFR-AT-0094_        ATGGGTGCCGCTTCTTTCACCGCCCAGGTCATCGTCCTCAACCACCCCGG  1049
F._fujikuroi_IMI_58289                ATGGGTGCCGCTTCTTTCACCGCCCAGGTCATCGTCCTCAACCACCCCGG  1049
F._solani_FSSC_5                      CTGGGTGCCGCCTCTTTCACCGCCCAGGTCATTGTCCTCAACCACCCTGG  1049
F._commune_MPI-SDFR-AT-0072           CAGGGTGCCGCTTCTTTCACCGCCCAGGTCATCGTCCTCAACCACCCCGG  1049
F._verticillioides_7600               ATGGGTGCCGCTTCTTTCACCGCTCAGGTCATTGTCCTCAACCACCCCGG  1049
F._graminearum_FGSG_08811T0           ATGGGTGCCGCTTCTTTCACCGCCCAGGTCATCGTCCTCAACCACCCCGG  1049
F._pseudograminearum_CS3096           ATGGGTGCCGCTTCTTTCACCGCCCAGGTCATCGTCCTCAACCACCCCGG  1049
AF-3_KC691533.1                       GTGAGCGT------------------GGT-----------ATCACC----  557
AF-3_KC691536.1                       GTGAGCGT------------------GGT-----------ATCACC----  557
Fusarium_spp._Strain_EI               GTGAGCGT------------------GGT-----------ATCACC----  520
                                        * * *                   ***           * ****

F._oxysporum_f._sp._lycopersic        TCAGGTCGGTGCTGGTTACGCTCCCGTCCTCGATTGCCACACTGCCCACA  1045
F._oxysporum_MPI-SDFR-AT-0094_        TCAGGTCGGTGCTGGTTACGCTCCCGTCCTCGATTGCCACACTGCCCACA  1099
F._fujikuroi_IMI_58289                TCAGGTCGGTGCTGGTTACGCTCCCGTCCTCGATTGCCACACTGCCCACA  1099
F._solani_FSSC_5                      CCAGGTCGGTGCCGGTTACGCCCCCGTTCTGGACTGCCACACTGCCCACA  1099
F._commune_MPI-SDFR-AT-0072           TCAGGTCGGTGCTGGTTACGCTCCCGTCCTCGATTGCCACACTGCCCACA  1099
F._verticillioides_7600               TCAGGTCGGTGCTGGTTACGCTCCCGTCCTCGATTGCCACACTGCCCACA  1099
F._graminearum_FGSG_08811T0           TCAGGTCGGTGCTGGTTACGCTCCCGTCCTCGATTGCCACACTGCCCACA  1099
F._pseudograminearum_CS3096           TCAGGTCGGTGCTGGTTACGCTCCCGTCCTCGATTGCCACACTGCCCACA  1099
AF-3_KC691533.1                       ----ATCG------------------------------ACATTGCCC---  570
AF-3_KC691536.1                       ----ATCG------------------------------ACATTGCCC---  570
Fusarium_spp._Strain_EI               ----ATCG------------------------------ATATTGCTC---  533
                                           ***                              * * *** *

F._oxysporum_f._sp._lycopersic        TTGCCTGCAAGTTCGCCGAGATCCAGGAGAAGATCGACCGCCGAACCGGT  1095
F._oxysporum_MPI-SDFR-AT-0094_        TTGCCTGCAAGTTCGCCGAGATCCAGGAGAAGATCGACCGCCGAACCGGT  1149
F._fujikuroi_IMI_58289                TTGCCTGCAAGTTCGCCGAGATCCAGGAGAAGATCGACCGCCGAACCGGT  1149
F._solani_FSSC_5                      TTGCCTGCAAGTTCGCCGAGATCCAGGAGAAGATCGACCGCCGAACTGGT  1149
F._commune_MPI-SDFR-AT-0072           TTGCCTGCAAGTTCGCCGAGATCCAGGAGAAGATCGACCGCCGAACCGGT  1149
F._verticillioides_7600               TTGCCTGCAAGTTCGCCGAGATCCAGGAGAAGATCGACCGCCGAACCGGT  1149
F._graminearum_FGSG_08811T0           TTGCCTGCAAGTTCGCCGAGATCCAGGAGAAGATCGACCGCCGAACCGGT  1149
F._pseudograminearum_CS3096           TTGCCTGCAAGTTCGCTGAGATCCAGGAGAAGATCGACCGCCGAACCGGT  1149
AF-3_KC691533.1                       ---TCTGGAAGTTCG-----------------------------------  582
AF-3_KC691536.1                       ---TCTGGAAGTTCG-----------------------------------  582
Fusarium_spp._Strain_EI               ---TCTGGAAGTTCG-----------------------------------  545
                                          *** *******

F._oxysporum_f._sp._lycopersic        AAGGCTACTGAGGCCGCCCCCAAGTTCATCAAGTCTGGTGACTCCGCCAT  1145
F._oxysporum_MPI-SDFR-AT-0094_        AAGGCTACTGAGGCCGCTCCCAAGTTCATCAAGTCTGGTGACTCCGCCAT  1199
F._fujikuroi_IMI_58289                AAGGCTACTGAGGCCGCCCCCAAGTTCATCAAGTCTGGTGACTCCGCCAT  1199
F._solani_FSSC_5                      AAGGCTGTTGAGTCCGCCCCCAAGTTCATCAAGTCTGGTGACTCCGCCAT  1199
F._commune_MPI-SDFR-AT-0072           AAGGCTACTGAGGCCGCCCCCAAGTTCATCAAGTCCGGTGACTCCGCCAT  1199
F._verticillioides_7600               AAGGCTACTGAGTCCGCCCCTAAGTTCATCAAGTCTGGTGACTCTGCCAT  1199
F._graminearum_FGSG_08811T0           AAGGCTACTGAGGCCGCCCCCAAGTTCATCAAGTCTGGTGACTCCGCCAT  1199
F._pseudograminearum_CS3096           AAGGCTACTGAGGCCGCCCCCAAGTTCATCAAGTCTGGTGACTCCGCCAT  1199
AF-3_KC691533.1                       -AGACTCCC------CGCT---------------------ACTATGTCAC  604
AF-3_KC691536.1                       -AGACTCCC------CGCT---------------------ACTATGTCAC  604
Fusarium_spp._Strain_EI               -AGACTCCT------CGCT---------------------ACTATGTCAC  567
                                       ** **         ***                      ***  * **

F._oxysporum_f._sp._lycopersic        CGTCAAGATGGTTCCCTCCAAGCCCATGTGTGTTGAGGCTTTCACTGACT  1195
F._oxysporum_MPI-SDFR-AT-0094_        CGTCAAGATGGTTCCCTCCAAGCCCATGTGTGTTGAGGCTTTCACTGACT  1249
F._fujikuroi_IMI_58289                CGTCAAGATGGTTCCCTCCAAGCCCATGTGTGTCGAGGCTTTCACTGACT  1249
F._solani_FSSC_5                      CGTCAAGATGGTTCCCTCCAAGCCCATGTGCGTTGAGGCTTTCACTGACT  1249
F._commune_MPI-SDFR-AT-0072           CGTCAAGATGGTTCCCTCCAAGCCCATGTGTGTTGAGGCTTTCACTGACT  1249
F._verticillioides_7600               CGTCAAGATGATTCCCTCCAAGCCCATGTGTGTTGAGGCTTTCACTGACT  1249
F._graminearum_FGSG_08811T0           CGTCAAGATGGTTCCCTCCAAGCCCATGTGTGTTGAGGCTTTCACCGACT  1249
F._pseudograminearum_CS3096           CGTCAAGATGGTTCCCTCCAAGCCCATGTGTGTTGAGGCTTTCACTGACT  1249
AF-3_KC691533.1                       CGTCAT--TGGT-------------ATGT-CGCTG-----TCC------T  627
AF-3_KC691536.1                       CGTCAT--TGGT-------------ATGT-CGCTG-----TCC------T  627
Fusarium_spp._Strain_EI               CGTCAT--TGGT-------------ATGT-TGTCG-----CTCA-----T  591
                                      *****   ** *             ****  *  *       *      *
```

```
F._oxysporum_f._sp._lycopersic      ACCCTCCTCTGGGTCGTTTCGCCGTCCGTGACATGCGACAGACTGTCGCC 1245
F._oxysporum_MPI-SDFR-AT-0094_      ACCCTCCTCTGGGTCGTTTCGCCGTCCGTGACATGCGACAGACTGTCGCC 1299
F._fujikuroi_IMI_58289              ACCCTCCTCTGGGTCGTTTCGCCGTCCGTGACATGCGACAGACTGTCGCC 1299
F._solani_FSSC_5                    ACCCCCCTCTGGGCCGTTTCGCCGTCCGTGACATGCGACAGACCGTTGCT 1299
F._commune_MPI-SDFR-AT-0072         ACCCTCCTCTGGGTCGTTTCGCCGTCCGTGACATGCGACAGACTGTCGCC 1299
F._verticillioides_7600             ACCCTCCTCTGGGTCGTTTCGCCGTCCGTGACATGCGACAGACCGTCGCC 1299
F._graminearum_FGSG_08811T0         ACCCTCCTCTGGGTCGTTTCGCCGTCCGTGACATGCGACAGACCGTCGCC 1299
F._pseudograminearum_CS3096         ACCCTCCTCTGGGTCGTTTCGCCGTCCGTGACATGCGACAGACCGTCGCC 1299
AF-3_KC691533.1                     CTCTCAATC-A---TGTCTCGCC--AC-TAACA-AT--CAA-CAGACGCC 666
AF-3_KC691536.1                     CTCTCAATC-A---TGTCTCGCC--AC-TAACA-AT--CAA-CAGACGCC 666
Fusarium_spp._Strain_EI             GCTTCATTCTA---CTTCTCTTCGTAC-TAACATAT--CACTCAGACGCT 635
                                           **       * **  *   * * ***     **  * *  **

F._oxysporum_f._sp._lycopersic      GTTGGTGTCATCAAGGCTGTCGAGAAGTCCAGCGGTGCTGCCGGCAAGGT 1295
F._oxysporum_MPI-SDFR-AT-0094_      GTTGGTGTCATCAAGGCCGTCGAGAAGTCCAGCGGTGCTGCCGGCAAGGT 1349
F._fujikuroi_IMI_58289              GTCGGTGTCATCAAGGCCGTCGAGAAGTCCACTGGTGCTGCCGGCAAGGT 1349
F._solani_FSSC_5                    GTCGGTGTCATCAAGGCCGTCGAGAAGGCTGCTGCTGGCTCCGGCAAGGT 1349
F._commune_MPI-SDFR-AT-0072         GTCGGTGTCATCAAGGCCGTCGAGAAGTCCGCTGGTGGTTCCGGCAAGGT 1349
F._verticillioides_7600             GTCGGTGTCATCAAGGCCGTCGAGAAGTCCTCTGGTGCTGCCGGCAAGGT 1349
F._graminearum_FGSG_08811T0         GTCGGTGTCATCAAGGCCGTCGAGAAGTCCACTGGCGCTGCTGGCAAGGT 1349
F._pseudograminearum_CS3096         GTCGGTGTCATCAAGGCCGTCGAGAAGTCCACTGGCGCTGCTGGCAAGGT 1349
AF-3_KC691533.1                     CCCGG--CCACC----------------------G--------------- 677
AF-3_KC691536.1                     CCCGG--CCACC----------------------G--------------- 677
Fusarium_spp._Strain_EI             CCCGG--TCACC----------------------GTGATTTCATCAAGAA 661
                                       **   ** *

F._oxysporum_f._sp._lycopersic      CACCAAGTCCGCTGCCAAGGCCGCCAAGAAAT-- 1327
F._oxysporum_MPI-SDFR-AT-0094_      CACCAAGTCCGCTGCCAAGGCCGCCAAGAAATAA 1383
F._fujikuroi_IMI_58289              CACCAAGTCCGCTGCCAAGGCCGCCAAGAAATAA 1383
F._solani_FSSC_5                    CACCAAGTCCGCTGCCAAGGCTGCCAAGAAGTAA 1383
F._commune_MPI-SDFR-AT-0072         CACCAAGTCCGCTGCCAAGGCCGCCAAGAAATAA 1383
F._verticillioides_7600             CACCAAGTCCGCTGCCAAGGCCGCTAAGAAAT-- 1381
F._graminearum_FGSG_08811T0         CACCAAGTCCGCTGCCAAGGCTGGTAAGAAA--- 1380
F._pseudograminearum_CS3096         CACCAAGTCCGCTGCCAAGGCTGGTAAGAAATAA 1383
AF-3_KC691533.1                     ----------------------------------
AF-3_KC691536.1                     ----------------------------------
Fusarium_spp._Strain_EI             CATGACTTGGGGT----AATCCCCCA-------- 683
```

Figure S3. Amino acid sequence alignment of GH12 enzymes.

Amino acid sequences of GH12 members were aligned using ClustalW. Identical amino acids are highlighted. Dots and colons indicate conserved amino acids with substitutions. Dashes indicate gaps. The 25-kDa protein (LC634256) from *Fusarium* spp. strain EI, CVL13720.1 from *F. proliferatum*, XP_018760412.1 from *F. verticillioides* 7600, XP_011325323.1 from *F. graminearum* PH-1, OXN20159.1 from *Aspergillus fumigatus*, and GAM41656.1 from *Talaromyces cellulolyticus* are shown. The putative active site is highlighted in yellow.

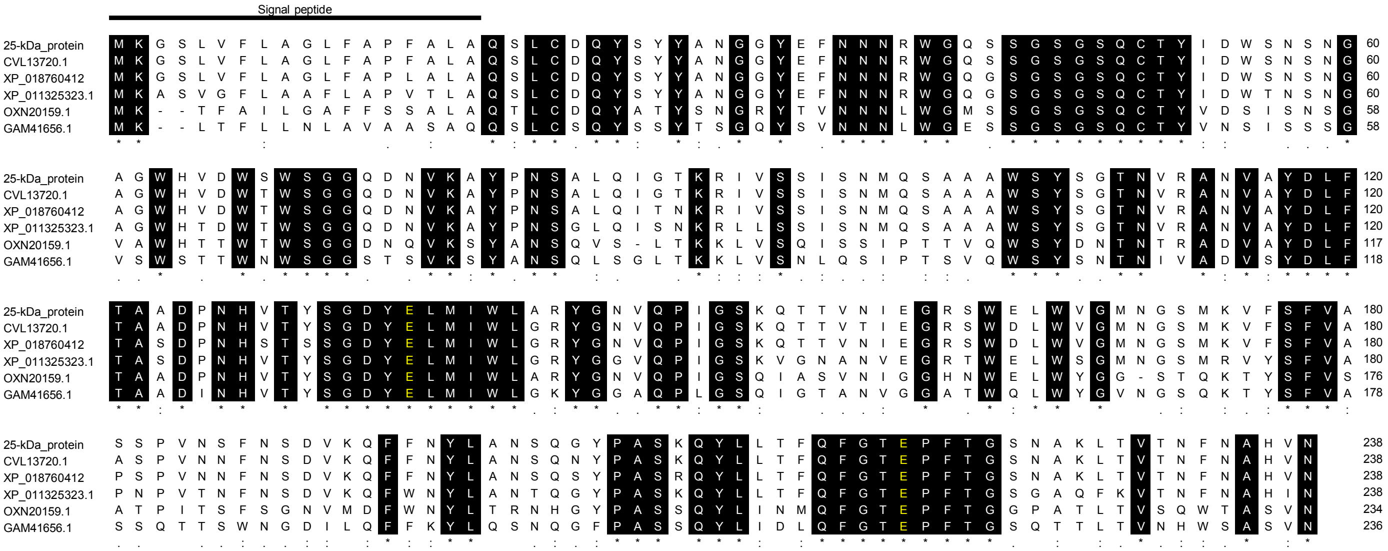

Supplement: Supplementary file 1 — Additional file 1: Table S1. Identification of the cellulolytic enzyme produced by Fusarium spp. strain EI. Table S2. Substrate specificity of the purified GH12 enzyme. Figure S1. Nucleotide sequence alignment of 18S rRNA. Figure S2. Nucleotide sequence alignment of EF-1α. Figure S3. Amino acid sequence alignment of GH12 enzymes. [file 13568_2020_1030_MOESM1_ESM.pdf]
